# Dotted collar placed around carotid artery induces asymmetric neointimal lesion formation in rabbits without intravascular manipulations

**DOI:** 10.1186/1471-2261-12-91

**Published:** 2012-10-17

**Authors:** Antti Kivelä, Juha Hartikainen, Seppo Ylä-Herttuala

**Affiliations:** 1 A.I.Virtanen Institute, University of Eastern Finland, Kuopio, Finland; 2 Department of Medicine, University of Eastern Finland, Kuopio, Finland; 3 Heart Center, Kuopio University Hospital, Kuopio, Finland; 4 Research Unit, Kuopio University Hospital, Kuopio, Finland

**Keywords:** Atherosclerotic lesion, Carotid collar, Rabbit, Shear stress

## Abstract

**Background:**

Neointimal formation in atherosclerosis has been subject for intense research. However, good animal models mimicking asymmetrical lesion formation in human subjects have been difficult to establish. The aim of this study was to develop a model which would lead to the formation of eccentric lesions under macroscopically intact non-denuded endothelium.

**Methods:**

We have developed a new collar model where we placed two cushions or dots inside the collar. Arterial lesions were characterized using histology and ultrasound methods.

**Results:**

When this dotted collar was placed around carotid and femoral arteries it produced asymmetrical pressure on adventitia and a mild flow disturbance, and hence a change in shear stress. Our hypothesis was that this simple procedure would reproducibly produce asymmetrical lesions without any intraluminal manipulations. Intima/media ratio increased towards the distal end of the collar with the direction of blood flow under macroscopically intact endothelium. Macrophages preferentially accumulated in areas of the thickest neointima thus resembling early steps in human atherosclerotic plaque formation. Proliferating cells in these lesions and underlying media were scarce at eight weeks time point.

**Conclusion:**

The improved dotted collar model produces asymmetrical human-like atherosclerotic lesions in rabbits. This model should be useful in studies regarding the pathogenesis and formation of eccentric atherosclerotic lesions.

## Background

Neointimal growth has been a subject for intensive research during several decades. A collar placed around a target vessel is one of the models that have been used to induce concentric intimal thickening in different animal species under macroscopically intact endothelium [1]. However, good models for asymmetric human-like lesion formation and neointimal growth are still lacking especially if endothelial denudation should be avoided. The role of adventitia in the neointimal formation has not been studied very much and interest has been merely focused on medial smooth muscle cells (SMC) and endothelium. However, new interest in the role of adventitia has emerged due to several findings concerning the role of adventitia in the production of reactive oxygen species (ROS) [2], expression of inducible nitric oxide synthase (iNOS) [3]), extracellular matrix production and degradation [4], neovascularization [5] and synthesis of various growth factors [5].

Changes in shear stress are important determinants of intimal hyperplasia [6,7]. Disturbed flow in curved regions and branches is proatherogenic, whereas laminar flow is atheroprotective. Laminar shear stress upregulates several genes involved in cell survival, cell cycle arrest, morphological remodelling and NO production [8]. Most models used for these studies have utilized isolated vessels in perfusion chambers, which is far from in vivo situation. Marano et al. used ligation of the right carotid artery thus causing high shear stress on the contralateral collared artery [9].

**Figure 1:**
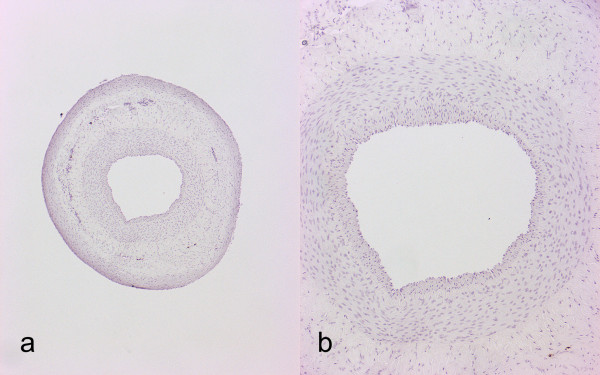
**Histological features of carotid artery eight weeks after hollow collar placement. a**) Magnification 4X. **b**) Magnification 10X. Neointima formation is modest and symmetric.

Collar models used previously have been based on symmetrical hollow collars [1]. In contrast to human atherosclerotic lesions this collar constantly produces a symmetric concentric intimal thickening (Figure 1). Here we describe a new collar model that reproducibly causes an asymmetrical eccentric neointimal thickening in rabbit carotid and femoral arteries.

## Methods

### Collar implantation

**Figure 2:**
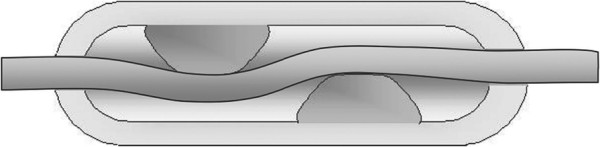
Design of the dotted collar.

New Zealand White rabbits (n=20, weight 3.2 to 4.4 kg) were used for the experiments. Rabbits were fed with normal rabbit chow enriched with 0.25% cholesterol for two weeks before any operation. General anaesthesia was induced using fentanyl-fluanisone (0.8 mg/kg + 2.2 mg/kg) intravenously and midazolam (1 – 1.5 mg/kg) intramuscularly. Left carotid artery and right femoral artery were exposed under local anaesthesia (lidocaine) and a dotted collar (Ark Therapeutics Oy, Kuopio, Finland) (Figure 2) was carefully placed around the vessel avoiding any operational damage. After the operation the wound was closed layer by layer with 5–0 Dexon. All experiments were approved by the Experimental Animal Committee of the University of Kuopio.

### Carotid flow measurement

Carotid flow velocities were measured at baseline before operation, acutely following the collar placement and at the end of the experiments at eight weeks using Acuson Sequoia C256 Echocardiography system with 15L8-S transducer at 14 MHz. Measurements were taken at an angle of 57-60°, using a colour doppler to guide pulsed doppler measurements to the area of peak flow. Peak velocity values were measured three times from each vessel and the highest value obtained was used for analyses.

### Histological analysis

Vessels were harvested eight weeks after the operation. Arterial segment inside the collars was divided into four equal parts (cranial, craniomedial, caudomedial, caudal). Paraffin sections were immersion-fixed in 4% paraformaldehyde/15% sucrose (pH 7.4) for 4 hours, rinsed overnight in 15% sucrose (pH 7.4), and embedded in paraffin. Neointima formation was measured after hematoxylin-eosin (HE) staining using Olympus AX70 microscope (Olympus Optical, Japan) with Analysis imaging software (Soft Imaging Systems GmbH, Germany). Lumen, internal elastic lamina and external elastic lamina were traced and lumen area and trace lengths were recorded. Immunohistochemical stainings were done using the following antibodies: CD31 (endothelium, dilution 1:50, DAKO), RAM-11(macrophages, dilution 1:200, DAKO), HHF-35 (smooth muscle cells [SMCs], dilution 1:50, DAKO). An avidin-biotin–horseradish peroxidase system was used for signal detection (Vector Elite Kit). Controls included stainings with class and species matched first antibodies and incubation where the first antibodies were omitted [10].

### Statistical analysis

Statistical analysis was performed with SPSS version 10.0.5 software package. After testing for normality of the data, Mann–Whitney U test was used for a pairwise (caudal vs. other segments in carotid arteries, cranial vs. other segments in femoral arteries) comparison of the collared segments. p <0.05 was considered statistically significant.

## Results

**Figure 3:**
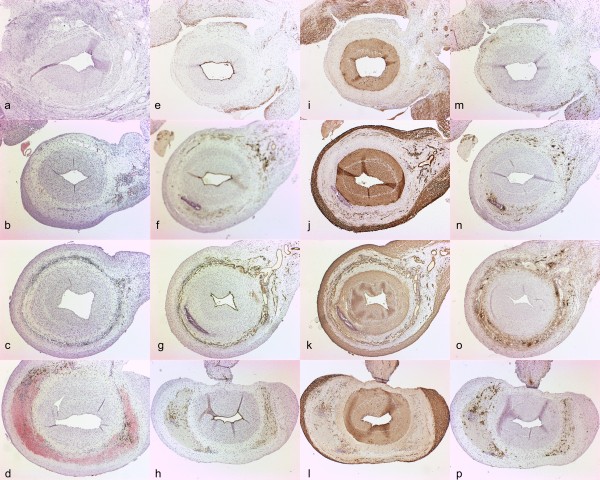
**Histological and immunohistochemical features of the lesions eight weeks after the placement of collar in carotid arteries. a** – **d**) Hematoxylin-eosin staining. **e** – **h**) Endothelial staining with CD31. Note intact endothelium. **i** – **l**) Smooth muscle cell staining with HHF-35. **m** – **p**) Macrophage staining with RAM-11. Note accumulation of macrophages in the asymmetric plaque. Original magnifications in all sections 4X.

**Figure 4:**
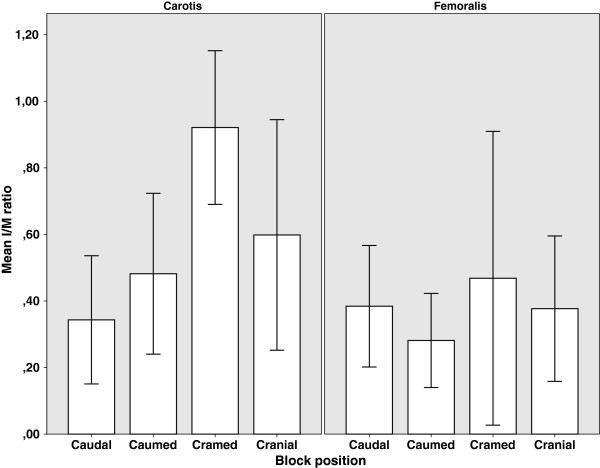
**Intima/media ratios in carotid and femoral sections.** Pooled data of all carotid and femoral sections. Intima-media (I/M) ratios in different segments of the collared carotid and femoral arteries. Values represent mean ± SEM. * p=0.002 compared to the caudal segment of the carotid artery. Direction of blood flow in carotid samples is from left to right and in femoral samples from right to left.

**Figure 5:**
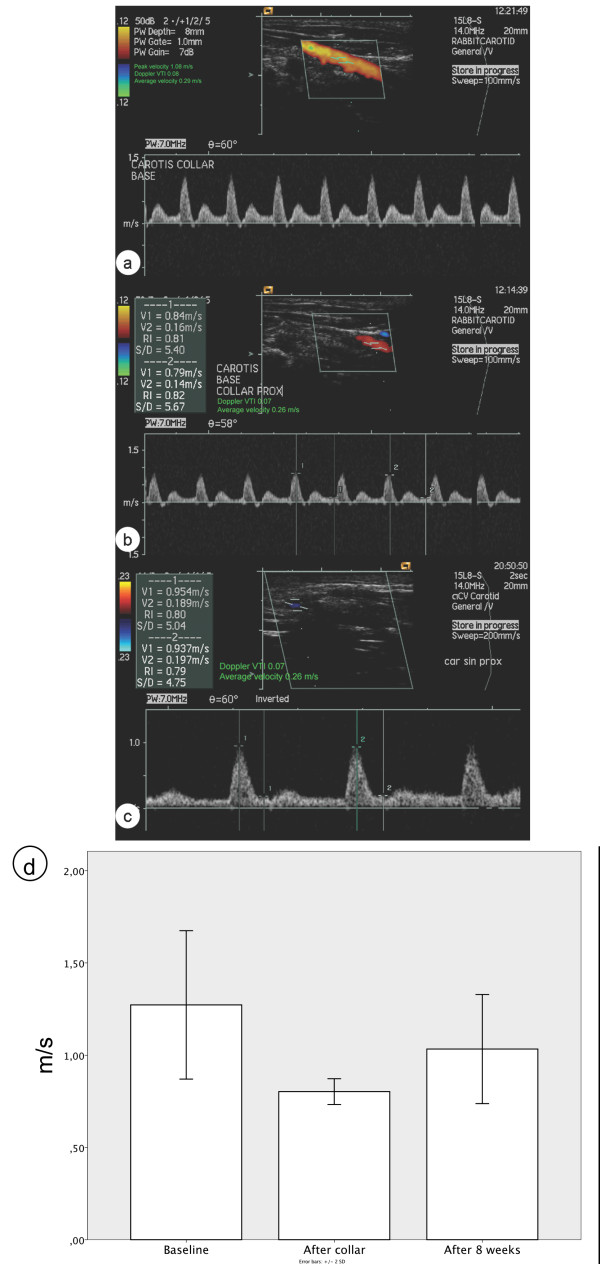
**Blood flow curves in the carotid arteries after the placement of collar. a**) Flow at baseline. **b**) Flow proximal to the collar at day 0 after the collar implantation. **c**) Flow proximal to the collar at eight weeks. **d**) Flow rate means at time points **a**-**c**, shown as m/s with 2 standard deviations.

After eight weeks, neointimal formation was detected in the collared arteries. Contrary to the previously used rabbit carotid collar models [1,11,12] predominantly asymmetric neointimal formation was observed, which in several cases resembled human lesions (Figure 3). Another new feature in this model was the gradual increase in the neointimal thickening along the flow direction in carotid segments. Intima/media ratio increased towards the distal end of the collar being 0.34 ± 0.09 in caudal segments, 0.48 ± 0.12 in caudomedial segments, 0.92 ± 0.38 in craniomedial segments (p = 0.002 vs. caudal) and 0.59 ± 0.17 in cranial segments. However, such findings were not seen in femoral arteries. (Figures 4, 5.)

**Figure 6:**
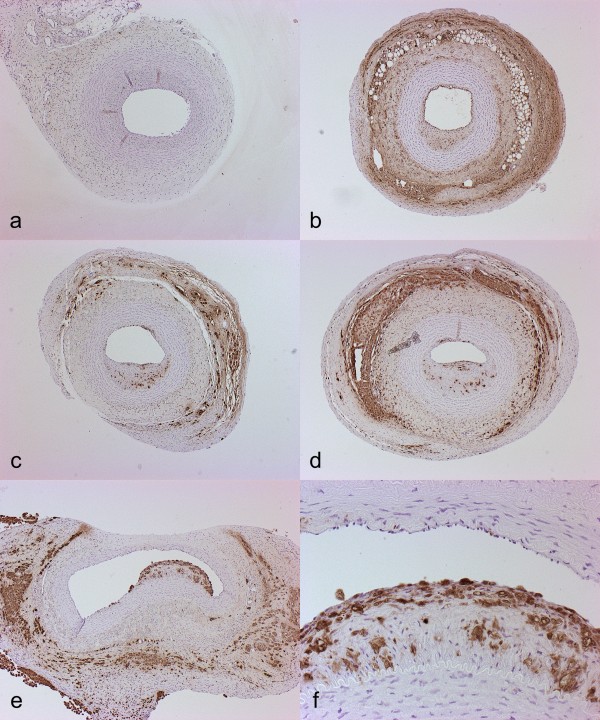
**Asymmetric intima formation in the direction of blood flow.** At eight weeks, statistically significant asymmetric neointimal growth was seen in the carotid arteries along the direction of blood flow. **a**) caudal, **b**) caudomedial, **c**) craniomedial, **d**) cranial. **e**-**f**) Representative sections of plaque formation, caudomedial, femoral artery. Original magnification in **a**-**e**) 4X, in **f**) 20X. RAM-11 staining.

Effect of the dotted collar on the velocity of the carotid artery blood flow was measured with Doppler ultrasound. Attenuation of the peak flow velocity in the carotid arteries was seen after the placement of the collar. However, the attenuation was modest and returned to normal levels after eight weeks follow-up despite of the development of neointima. In native, non-operated carotids blood flow proximal to the site of the collar was 1.27±0.20 m/s, blood flow immediately after the collar placement was 0.77±0.06 m/s. The corresponding value eight weeks after the collar implantation was 1.17±0.21 m/s. (Figure 6).

Immunohistochemical analysis showed macrophage accumulation in areas of the thickest neointimal formation and in adventitia (Figures 3 m-p; 5). In some collared vessels the density of macrophage accumulation also increased with the increasing neointimal area and according to the blood flow (Figure 5 a-d). The number of macrophages in the lesions varied between 5 to 20 cells/100 μm^2^. Intact endothelium was present at eight weeks in almost all segments (Figure 3 e-h). Endothelial layer was always present in areas of large, plaque-like lesions. In a few sections, some local detachment of endothelial cells was noted, but this did not correlate with the increase in the neointimal thickening, and was mainly considered as a sporadic finding. The bulk of the lesions consisted of SMC as shown by α-actin immunostaining (Figure 3 i-l). In some lesions, accumulation of macrophages was also seen in the deep layers of the lesions (Figure 5 c,d,f) resembling early stages in atheroma formation. Lesion neovascularization could be seen increasing towards cranial (distal) segments in a similar fashion as plaque thickness, as detected by CD31 immunostaining (Figure 3 e-h).

## Discussion

Molecular and cellular factors leading to neointimal growth include proliferation and migration of vascular SMC, matrix remodelling, lipid accumulation, LDL oxidation, endothelial dysfunction and inflammatory reactions [13]. Physical determinants proposed to explain neointimal growth in the collar model are changes in shear stress, occlusion of vasa vasorum thus affecting oxygen balance and metabolic status, loss of perivascular innervation and twisting of the collared artery [11]. Marano et al. showed that increased shear stress was protective against neointimal thickening in rabbit carotid collar model [9]. However, they used hollow collars with an even inner surface as originally developed by Booth et al. [1]. In the new model our intention was to alter the normal laminar flow to a turbulent one by twisting the vessel thus giving differential pressure and flow signals on the endothelium.

Vascular adventitia is known to be activated in a variety of cardiovascular diseases. It has been postulated that adventitial fibroblasts proliferate and migrate into the neointima in response to injury [2,14]. It has also been shown that direct adventitial injury in the absence of endothelial damage is sufficient to cause neointimal lesions [15]. Barker et al. [16] removed adventitia in hypercholesterolemic rabbits, which induced intimal hyperplasia and macrophage accumulation. Our dotted collar imposed an altered pressure on adventitia and we hypothesized that this is sufficient to disturb normal homeostasis in the vessel wall and cause lesion formation. It was found that dotted collar induced the formation of asymmetric SMC-rich lesions that also accumulated macrophages and started to resemble human-like lesions.

The effect of the collar placement in femoral versus carotid arteries has not been studied before. In this series a clear difference in the reactivity to the dotted collar was seen between carotid and femoral arteries: collar-induced neointimal thickening in the femoral arteries was only mildly increased along the direction of the blood flow and did not reach statistical significance. We consider this difference in the neointimal development probably as a result of the differential adventitial pressure. However, differences in the anatomical locations of the arteries may also have contributed to these findings. Limitations of this study include limited technical details in ultrasound acquisition: in future with intra-arterial flow probe the turbulence inside the dotted collar can be studied and correlated to the neointimal changes. Also, collar device can be used for local drug, protein and gene delivery [12,17,18], which should allow further evaluation of the pathogenetic mechanisms under macroscopically intact endothelium.

## Conclusions

In conclusion, the dotted collar model causes asymmetrical neointimal lesion formation with the accumulation of macrophages. This model should be useful for studies addressing the pathogenesis of neointimal SMC accumulation and the formation of eccentric atherosclerotic lesions in large arteries.

## Competing interests

The authors declare that they have no competing interests.

## Authors’ contributions

AK carried out all animal experiments and most tissue analyses. He also participated in the drafting of the manuscript. JH carried out ultrasound analyses and participated in the drafting of the manuscript. SY-H participated in the design of the study, gave oversight for the entire study and drafted and finalized the manuscript. All authors read and approved the final manuscript.

## Pre-publication history

The pre-publication history for this paper can be accessed here:

http://www.biomedcentral.com/1471-2261/12/91/prepub
